# Commonalities and differences in the implementation of models of care for arthritis: key informant interviews from Canada

**DOI:** 10.1186/s12913-016-1634-9

**Published:** 2016-08-19

**Authors:** Cheryl A. Cott, Aileen M. Davis, Elizabeth M. Badley, Rosalind Wong, Mayilee Canizares, Linda C. Li, Allyson Jones, Sydney Brooks, Vandana Ahlwalia, Gillian Hawker, Susan Jaglal, Michel Landry, Crystal MacKay, Dianne Mosher

**Affiliations:** 1 Department of Physical Therapy, Faculty of Medicine, University of Toronto, Toronto, Canada; 2 Arthritis Community Research & Evaluation Unit and Division of Health Care and Outcomes Research, Toronto Western Research Institute, University Health Network, Toronto, Canada; 3 Institutes of Health Policy, Management and Evaluation and Rehabilitation Science, Faculty of Medicine, University of Toronto, Toronto, Canada; 4 Dalla Lana School of Public Health, Faculty of Medicine, University of Toronto, Toronto, Canada; 5 Department of Physical Therapy, Department of Medicine, University of British Columbia and Arthritis Centre of Canada, Vancouver, BC Canada; 6 Department of Physical Therapy, Faculty of Rehabilitation Medicine and School of Public Health, University of Alberta, Edmonton, AB Canada; 7 Ontario Division of The Arthritis Society, Toronto, ON Canada; 8 Ontario Rheumatology Association, Toronto, Canada; 9 Women’s College Hospital and the University of Toronto, Toronto, ON Canada; 10 Department of Physical Therapy, University of Toronto, Toronto, ON Canada; 11 Doctor of Physical Therapy Division, Department of Community and Family Medicine, Duke University Medical Centre, Durham, NC USA; 12 Health Care and Outcomes Research, Toronto Western Research Institute, University of Toronto, Toronto, ON Canada; 13 Arthritis Alliance Canada, Vancouver, BC Canada

**Keywords:** Models of care, Arthritis, Total joint replacement

## Abstract

**Background:**

Timely access to effective treatments for arthritis is a priority at national, provincial and regional levels in Canada due to population aging coupled with limited health human resources. Models of care for arthritis are being implemented across the country but mainly in local contexts, not from an evidence-informed policy or framework. The purpose of this study is to examine existing models of care for arthritis in Canada at the local level in order to identify commonalities and differences in their implementation that could point to important considerations for health policy and service delivery.

**Methods:**

Semi-structured key informant interviews were conducted with 70 program managers and/or care providers in three Canadian provinces identified through purposive and snowball sampling followed by more detailed examination of 6 models of care (two per province). Interviews were transcribed verbatim and analyzed thematically using a qualitative descriptive approach.

**Results:**

Two broad models of care were identified for Total Joint Replacement and Inflammatory Arthritis. Commonalities included lack of complete and appropriate referrals from primary care physicians and lack of health human resources to meet local demands. Strategies included standardized referrals and centralized intake and triage using non-specialist health care professionals. Differences included the nature of the care and follow-up, the role of the specialist, and location of service delivery.

**Conclusions:**

Current models of care are mainly focused on Total Joint Replacement and Inflammatory Arthritis. Given the increasing prevalence of arthritis and that published data report only a small proportion of current service delivery is specialist care; provision of timely, appropriate care requires development, implementation and evaluation of models of care across the continuum of care.

**Electronic supplementary material:**

The online version of this article (doi:10.1186/s12913-016-1634-9) contains supplementary material, which is available to authorized users.

## Background

The management of arthritis is increasingly an issue for Canadian jurisdictions due to factors including, but not limited to, the aging of the population and an increasing prevalence of chronic disease in a time of economic constraint. The 2005 Summit on Standards for Arthritis Prevention and Care identified access to appropriate and timely care as a right and a priority for people with arthritis [1]. Given the prevalence of arthritis (1 in 6 Canadians now have some form of arthritis) and that the prevalence is expected to increase by more than 50 % by 2020 [2] due to population aging and increasing rates of obesity coupled with limited health human resources to treat people with arthritis and musculoskeletal conditions, access to care issues are likely to continue to dominate the health care agenda. The perceived imbalance between supply and demand has ignited the policy debate in Canada’s primarily publicly funded health system regarding the best ways in which to provide timely access to health services for people with arthritis.

Health policy and decision makers continue to look for alternative ways to optimize access to and delivery of quality care. Professional organizations (e.g. Canadian Orthopaedic Association, Canadian Rheumatology Association, Arthritis Health Professionals Association, Arthritis Alliance of Canada etc.) have identified models of care for arthritis as a priority and funding agencies such as the Canadian Institutes of Health Research Institute of Health Services [3] and Policy Research and The Arthritis Society [4] have stated research priorities in the development and evaluation of innovative models of care for arthritis. Given these agendas, we need an understanding of the landscape of existing models of care for arthritis such that future work can address gaps and opportunities and models of care can be developed, implemented and evaluated to maximize care and outcomes for people with arthritis.

Important policy and clinical activity to enhance access is already underway across Canada. The identification of reduction of wait times for hip and knee replacement as a priority in the 2004 Health Accord [5] and the transfer of federal dollars to the provinces to reduce wait times created the impetus for the development of innovative models of care for arthritis to improve access and care for this patient group over the past decade. While some models of care for conservative management of arthritis have existed for decades, many new approaches to improve timely care, particularly for people with early inflammatory disease, also have developed, particularly since the advent of Disease Modifying AntiRheumatic Drugs (DMARDs). Yet, timely access to these treatments remains a priority at national, provincial, and regional levels, as both policy makers and the arthritis health care community seek alternative, effective models of health care delivery. With the exception of hip and knee total joint replacement (TJR) which has taken a provincial [6–8] and national approach [9], most models of care for arthritis have been implemented in local contexts [10, 11].

The term “models of care” is used with many different meanings encompassing condition-specific care to service delivery [12]. It has been defined as “an evidence-informed policy or framework that outlines the optimal manner in which condition specific care should be made available and delivered to consumers” [12]. This definition and approach suggests a top-down approach to the development of models of care yet at the time of this research the range of models of care for arthritis in Canada have most often been developed at the local level [13]; however, these local contextual factors have not been examined in-depth.

Consideration of local contextual factors in studies of health service delivery can include factors such as population health needs, geography, provider supply, technology and local and organizational policies [14]. In addition, local contextual factors can be influenced by national, provincial and local policies, health care system organization, and payment and incentive systems [15]. Some of these local contextual considerations may not be amenable to traditional evaluative approaches, yet could point to important contextual considerations with respect to health policy and service delivery. The purpose of this study is to examine existing models of care for arthritis in Canada at the local level in order to identify contextual commonalities and differences in their implementation.

## Methods

### Setting

Canada has federally mandated publicly funded health care; however, the implementation of health care is a provincial responsibility [16]. Further, the Canada Health Act only stipulates universal coverage of physician and in-hospital health services [17]. Funding for community-based, non-physician services outside of hospitals is determined provincially.

The study was conducted in three Canadian provinces: British Columbia; Alberta; and, Ontario. Ontario is the largest province with a population of over thirteen million, whereas British Columbia and Alberta have more similar population sizes (approximately 4 million each). These provinces were chosen as: 1) they had significant activity related to innovative approaches to arthritis care; 2) they represent geo-political differences in delivery of health care; 3) they all have vast areas with low population density; 4) they have differences in health human resource availability for provision of arthritis care [18]; 5) the scope of practice varies (and is evolving) for physiotherapists, occupational therapists and pharmacists who are often involved in arthritis care [19]; and, 6) service availability and coverage by provincial health plans vary (e.g. Ontario has some community-based rehabilitation services specifically for persons with arthritis that are paid for via provincial health care plans where there is currently no similar service in British Columbia or Alberta). As such, the models in these provinces represent a diverse base in which models of care for arthritis have been developed and implemented across the country.

### Study design

We used a two phase qualitative descriptive approach [20] beginning with: key informant interviews with program managers and/or care providers of arthritis service delivery; followed by more detailed examination of 6 models of care (two in each province). The purpose of the first phase was to identify the scope of existing models of care. The purpose of the second phase was to explore in more depth the strategies currently being utilized in various settings to address issues identified in the first phase such as lack of complete and appropriate referrals from primary care providers and lack of health human resources to meet local demand, particularly in rural and remote areas.

### Type of participants

- Phase 1: Key informant interviews
- A purposive sample of key informants included participants who represented various types of arthritis models of care and/or who were known opinion leaders in arthritis care delivery. Recruitment occurred through: our research team members; existing contacts including polling national participants from our workshop *Meeting the Challenges of Arthritis*: *Think Tank on Extended Roles for Rehabilitation Professional* [21] to ensure that we identified programs appropriate for people with arthritis; and, through the use of a snowball technique whereby key informants who were interviewed identified other models and/or individuals who could inform the study. Key informants were selected to ensure representation of professions, practice sectors, settings and geographic variation. Whenever possible, we interviewed more than one stakeholder from an identified model of care to ensure depth of information and varying perspectives to confirm findings. Data collection ceased once we were no longer identifying any additional types of models.
- Phase 2: In-depth case studies
- Sampling for the case studies was done from the models of care for arthritis identified in Phase 1. Our initial goal was to identify two cases in each province, one in a more urban setting and the other rural, for a total of three rural and three urban. However, in one province (British Columbia), models of care for arthritis in rural/remote areas are highly integrated with centralized service delivery involving the province’s largest city, Vancouver. Therefore we chose two rural areas for the case studies as they each incorporated the urban setting.
  Co-investigators from the respective provinces as well as key informants from Phase 1 identified the key players who would be relevant to provide the information we required for our in-depth interviews. In addition, a second interview was completed with those key informants from Phase 1 that held/described having a decision-making role/position or who identified themselves as key in the development of the model of care for arthritis in order to identify important team members/key informants in each case study.

### Data collection and analysis

Key informants were interviewed over the telephone by a single interviewer (RW) using a semi-structured interview schedule developed in consultation with research team members (Additional files 1 and 2). Potential participants were sent a Consent Information Letter prior to the interview with verbal consent obtained prior to initiating the telephone interview (Additional files 3 and 4). At the beginning of each interview, participants were asked to identify their job titles and roles, their educational backgrounds, their years of experience and the setting of the model. They then responded to a general question about how care was organized for people with arthritis in their setting. Probes were used as necessary related to the target patient group, health care providers involved in care, and service access. On average, interviews were one hour. Memoing was conducted during the course of and at the end of the interview by the interviewer.

Interviews were recorded, transcribed verbatim by a professional transcriptionist, and imported into NVivo 9 software for analysis using a qualitative descriptive approach [20]. The data were analyzed as follows: two research assistants (RAs) and two of the investigators (AMD, CC) independently coded three transcripts then met to discuss emerging themes and concepts and develop the coding scheme. All subsequent interviews were independently coded and compared to ensure consistency in coding between the two RAs. Any discrepancies in coding were resolved through discussion.

The investigators and RAs met frequently to discuss the unfolding analysis. As the interviews progressed it became clear that there were two different sub-groups present in the identified models of care, even though in some smaller centres the health care professionals may overlap between them. These models of care addressed Total Joint Replacement (TJR) and Inflammatory Arthritis. Flow charts and team “maps” were developed for each of the six case studies for TJR and Inflammatory Arthritis from which we developed overall summary maps for TJR and Inflammatory Arthritis. As the analysis progressed, teleconferences were held with co-investigators from each province to ensure accuracy of findings. Research findings were then presented and discussed in teleconferences or face-to-face meetings with the entire research team. At no time did the research team attempt to compare effectiveness or utility of the models of care either within or between provinces, rather we focused on identifying commonalities and differences in how different provinces addressed common issues encountered.

## Results

Seventy individual key informant interviews were conducted in Phase 1 between July 2010 and September 2012 (22 in British Columbia; 20 in Alberta, and 28 in Ontario). These key informant participants included 67 clinicians with some having a director/manager or coordinator role and three additional directors who had no health care professional certification. Professional representation included 24 physiotherapists, 5 occupational therapists, 10 nurses/nurse practitioners, 1 each from psychology and social work, 5 primary care physicians, 13 rheumatologists, and 8 orthopaedic surgeons. The key informants had between 1 and 36 years of experience related to arthritis management and care (average 19.4 years). Of the 70 interviews, the key informants represented 40 different locations of care delivery.

In Phase 2, twenty-eight participants were interviewed in the six case studies (9 in British Columbia, 10 in Alberta and 9 in Ontario). These interviews were conducted between November 2011 and January 2013. Of the 28 interviewees: 7 had a provincial program/health region management role; 2 a health region professional practice role; 10 had an institution/program level management role; and, 9 were care providers with a triage, coordination, system development role.

The case studies with rural populations varied in size: Thunder Bay, Ontario, 154,067; Prince Rupert, British Columbia, 13, 052; Kootenay, British Columbia, 75,000; Edson/Hinton, Alberta, (two different communities but within one health region) each with a population of approximately 9,000. London, the urban setting in Ontario, had a population of approximately 425,000 whereas the urban setting in Alberta (Calgary) had a population of 1.4 million. All case study locations had a similar overall incidence of arthritis (approximately 20 %) except for the urban area in Alberta (13 %) [22]. Overall Alberta has a younger population than Ontario or British Columbia, probably due to the oil industry, which may explain the lower prevalence of arthritis.

As the data analysis progressed it became apparent that although participants were talking about models of care for arthritis, they were actually talking about two sub-groups of patients: 1) patients with end stage osteoarthritis (OA) being considered for TJR; and, 2) patients with Inflammatory Arthritis. The models of care for these groups differed, even though they may all be seen in the same setting and sometimes by the same health care professionals, depending on local contextual issues such as the availability of health care professionals and the size of the community. We identified a number of common factors that impacted implementation at the local level. These common factors affected models of care for both TJR and Inflammatory Arthritis but for different reasons given the underlying disease trajectory of the two conditions.

The results are presented as follows: first we describe the traditional models of care for TJR and Inflammatory Arthritis; second, we identify commonalities in how the traditional models of care for arthritis have been modified in different contexts to address local needs; and, third, we identify differences in the models of care with respect to TJR and Inflammatory Arthritis.

### Traditional model of care for arthritis

We identified an underlying *traditional model of care for arthritis* that was the basis upon which the innovations and changes to service delivery were implemented by the participants. In this traditional model of care, after seeking care from a primary care physician, an individual is referred to a medical specialist (e.g. a rheumatologist or orthopaedic surgeon) for diagnosis, consultation and management. The overall care of the patient is then transferred back to the primary care physician with periodic review by the specialist.

The traditional model of care for TJR involves the primary care physician sending a referral to the surgeon. The surgeon then sees the patient and, if considered appropriate, schedules surgery. All patients are seen by the surgeon whether or not they ultimately require surgery. It is estimated that using this model of care, only about 20 % of the patients who end up seeing a surgeon actually receive surgery [23]. Following surgery patients may be discharged to subacute, inpatient rehabilitation or home with or without home care or out-patient physiotherapy. Once discharged from hospital, if there are no post-operative complications, they are typically followed by the surgeon 1–3 times over the year post-surgery.

There are two main phases in the traditional model of care for TJR where wait times are an issue: from the time of the referral from the primary care physician to actually having a surgical consult; and from the time of the surgical consult to actually having the surgery. The issues of time from surgical consult to surgery were addressed by tackling inefficiencies in the system such as more efficient use of consults with anesthesiology and internal medicine, pre-operative home visits to facilitate acute hospital discharge, and better through put in operating rooms (e.g. two operating rooms running simultaneously with one surgeon). By the time we conducted our interviews, most of these issues had already been addressed by the surgical wait time strategy of each province and the current focus of the models of care was on the first phase: referral from the primary care physician to surgical consult.

The traditional model of care for Inflammatory Arthritis is similar to that for TJR in that patients are referred by primary care physicians to the rheumatologist who initiates medical management that is continued by the primary care physician with the patient followed by the rheumatologist as needed. There are three major areas where there are likely delays in access to rheumatology care: 1) symptom onset and assessment in primary care; 2) first visit to a primary care physician and rheumatology referral; and, 3) time waiting to see the rheumatologist [24]. Any delay in seeing a rheumatologist is increasingly an issue due to the need for aggressive early intervention with DMARDs and biologic agents to improve clinical outcomes, functional status and quality of life [24].

In contrast to TJR where the process of care culminates in patients accessing surgery in a timely model, in the traditional model of care for Inflammatory Arthritis, the focus is on getting the patients with the greatest acuity to the rheumatologist in a timely manner to initiate an appropriate treatment plan. The rheumatologist provides the diagnosis and institutes appropriate medical management. Often these models of care are established in a disease specific context e.g. early rheumatoid arthritis, psoriatic arthritis, scleroderma, ankylosing spondylitis, lupus, complex osteoarthritis (OA). Hence, these models of care for arthritis require referral to a rheumatologist and determination or confirmation of at least a provisional diagnosis for access. In most cases, the rheumatologist is the entry point to accessing teams of other arthritis health care professionals.

### Commonalities in implementation of models of care for arthritis

Although the overall purpose of the models of care for TJR and Inflammatory Arthritis differ due to differences in disease trajectory, at the local level, common issues such as lack of complete and appropriate referrals from primary care physicians and lack of health human resources to meet local demand were identified as affecting the development and implementation of both models of care.

1. Lack of complete and appropriate referrals
  In Canada, access to medical specialists generally requires a referral from a primary care physician. For both the models of care for TJR and Inflammatory Arthritis issues of incomplete or inappropriate referrals from primary care physicians were identified as having major impacts on the implementation of efficient service delivery.
  For TJR, most of the models of care addressed the issue of appropriateness of the surgical referral by: 1) ensuring that the referral from the primary care physician contained all the appropriate information and that all the necessary tests, x-rays etc. had been completed prior to the patient seeing the surgeon; and 2) determining which of the referred patients were potentially appropriate surgical candidates prior to seeing the surgeon. According to one informant,

“*There were patients sitting on surgical consult wait lists that shouldn’t be there, that were far too early. But the GP (General practitioner) just didn’t know what else to do with them.”*

#### Standardized referrals

Many of the TJR models of care had implemented the use of standardized referrals to address issues of incomplete or inappropriate referrals from primary care physicians. Not only appropriateness as to whether patients were surgical candidates but also which surgeon is appropriate (not all surgeons perform both hip and knee TJRs). These standardized referrals are mainly completed by primary care physicians, but some specialists in Alberta also accept referrals from primary care nurses and physiotherapists.

*“There is a standardized referral form so that all the information is what is required by the people that are screening the referral…diagnostic tests that are required both from a lab and a diagnostic imaging perspective are included.”*

As with the TJR model of care, the need to standardize and screen referrals from primary care physicians to rheumatologists for Inflammatory Arthritis was identified as a key priority. As with TJR, Inflammatory Arthritis paper referrals are screened to ensure referrals are appropriate and information is complete for accurate prioritization, however, the information requested on the standardized rheumatology referral forms differ slightly from that for TJR. The rheumatology referral forms ask about inflammatory markers, morning stiffness, swollen joints, and other systemic features that would particularly identify those with new onset or inflammatory arthropathies requiring urgent consult by the rheumatologist. The goal is to gather adequate information so that when the rheumatologist sees the patient a diagnosis can be made and treatment can be initiated in a timely fashion.

#### Central intake/Pooled referrals

In addition to standardized referrals, many of the models of care had implemented central intake/pooled referral systems whereby all referrals were sent to a central intake where they were screened/triaged by 1) paper prioritization and/or 2) an in-person assessment.

#### Paper prioritization screening

The purpose of paper prioritization was to identify the provisional diagnosis and ensure that all the necessary information had been provided. If so, the referral was then sent to the appropriate practitioner, for example an orthopaedic surgeon or a rheumatologist.

*“If we can screen patients at the very beginning to know which people are surgical and which are non-surgical then our ultimate goal would be to only send potential surgical candidates on to see the surgeon. What that does is it increases the productivity of the surgeon because they’re really focused on providing the type of care that is required.”*

In Inflammatory Arthritis models of care, referrals may also be sorted to ensure patients are seen in the most appropriate program/clinic and in a timely fashion, so equally distributing workload and avoiding long wait times. Examples include an early arthritis/intervention clinic, programs for specific diseases such as lupus, and first available rheumatology nurse practitioner’s or rheumatologist’s clinic. Paper triage might be conducted by the rheumatologist, a nurse, and/or by a physiotherapist with specialty training. The referrals are reviewed, additional information including diagnostics requested, and prioritized based on urgency level. All referrals that suggest Inflammatory Arthritis are seen by the rheumatologist.

#### In-person screening assessments

In addition to paper prioritization, in-person screening assessments prior to seeing the specialist may be performed by a variety of health care professionals depending on the local context. For example, Extended Role/Extended Scope Practitioners (usually physiotherapists or occupational therapists) are used in Ontario whereas in Alberta, the role may be done by a retired orthopaedic surgeon or a primary care physician with a musculoskeletal specialty. In British Columbia, some primary care physicians have developed enhanced skills such that they can provide conservative management or triage people who are candidates to the orthopaedic surgeon (e.g. for foot and ankle surgery). In some larger settings in-person screening assessments are conducted by interdisciplinary teams involving physiotherapy, occupational therapy and nursing. Often these Extended Role therapist or nurse-led clinics are held in parallel with the orthopaedic surgeon’s clinic so that potential surgical candidates can be seen by the surgeon on the same day.

The value of screening is illustrated by the following participant who was asked what determines whether a patient will see the musculoskeletal screener or the orthopaedic surgeon:

*“Well it’s availability of both, and it’s reliant on funding as well…we were given some money to do some screening which of course dropped our wait list to be seen substantially. And that funding is no longer there. So what we do is we still continue to screen because really it makes our surgeons more efficient.”*

The purpose of the TJR screening assessments is to determine if the referred patient is a candidate to be seen by the surgeon for consideration of surgery. If not, they are referred back to their primary care physician with instructions to return if their symptoms get worse, or to another, more appropriate health care professional. Patients triaged to conservative management are those whose needs are considered non-surgical who may require some other form of treatment such as physiotherapy, weight loss, cortisone injections or bracing, or who may be deemed too early for surgical intervention.

*If the patient is non-surgical now because they have some medical problems…or they have a weight problem, then we would provide them with some recommendations about what they can do to get in better shape. Or to try to relieve some of the aches and pains that they have with their joints. We would provide that information to the patient and their family physician so that they know what has been recommended. Then if the patient requires further follow-up then they would come back to a central clinic where that would be provided or they would be cared for back in the community by their family physician.*

A few models of care try to work with these patients to develop ‘personalized care plans’ with the intent of helping patients understand what to do and how to access local resources. For these non-surgical, non-inflammatory disease patients, their ongoing care is now dependent on the funding and services available in their own community. Although recognized as important for models of care for arthritis, this component is often not well developed.

*..again I am talking about the surgical arthritis patients, not the patients with inflammatory arthritis or complex osteoarthritis that aren’t felt to be surgical candidates. That part of the model has never been fully developed, although it was in the plans originally.*

In contrast, within Inflammatory Arthritis models of care, the focus of screening is to identify those patients with the greatest acuity. In some settings in Ontario with Extended Role therapists, those referrals deemed as most likely to be non-inflammatory arthritis are triaged to The Arthritis Society therapist/Advanced Clinician Practitioner in Arthritis Care. These therapists would see these patients, provide an assessment, and develop a care plan eliminating the need for involvement of the rheumatologist. In situations where Extended Role therapists are available, they are viewed as valuable adjuncts to rheumatology specialist care. As one rheumatologist said,

*“So you can use those individuals as enhancers or extenders of your own physical practice. And these individuals are becoming more and more common in a variety of community settings where there are tremendous pressures on demand for those fewer physicians who are available. And this is a good thing because this provides expertise that can triage and allocate relatively scarce resources to the appropriate practitioner.”*

2. Lack of Health Human Resources to meet local demand
  There are a number of system and local macro issues that affect access to health human resources at the local level. At the system level, there is the overall shortage of specialists. At the local level these shortages are aggravated by geographic and population issues. Canada is a very large country, geographically, with the majority of the population clustered along the United States border. Although there are many large urban settings in Canada, there are still many Canadians who live in rural/remote areas with little access to specialists such as orthopaedic surgeons and rheumatologists. The local solutions that have evolved to address these issues focus on either: 1) getting the patients to the specialist; or 2) getting the specialist to the patients. As one respondent said,

*We have a surgeon that travels to …once a month and he does five hip replacements out there on the day that we go. And the patients that need to be seen by the surgeon are seen in between his surgical cases. And I (Extended Role physiotherapist) do the post-operative follow-up out there as well. And this spares (the patients) three and a half hours of driving each way for a 15 min follow-up appointment. We see anywhere from 25 to 40 patients per day.*

Thus, in smaller communities, services are cyclical and rely on the amount of time health care professionals have and the availability of resources. For example, telemedicine might be useful to mitigate travel, but health care professionals reported limited time to apply for funding for such programs, work out a program, devise policies and procedures, and to coordinate with the specialist to enable the service to happen.

The province of British Columbia provides an example of the local context of geography and demographics. British Columbia is a very mountainous, coastal province with a small population scattered over a variety of towns and islands. As described earlier, models of care for arthritis in British Columbia all involve centralized care in the city of Vancouver. Although there may be various reasons for this, one is the impact of geography. All flights within the province go into and out of Vancouver therefore making it logical to send patients from smaller communities to Vancouver as they would have to fly there anyway to connect to other locations. Therefore, specialist services in British Columbia are more centralized than in the other two provinces.

### Differences in implementation of models of care for arthritis

It is when we shift to examine the actual care provided in TJR and Inflammatory Arthritis models of care that differences between the two emerge, particularly in terms of interventions provided, follow-up, role of the specialist, involvement of other arthritis health care professionals and location of service delivery (see Table 1).

**Table 1:** Differences in TJR and inflammatory arthritis models of care

	TJR	Inflammatory arthritis
Triage	Appropriateness for surgery	Medical Acuity
Intervention	Single Episode of Care (Surgery)	Multiple Episodes of Care for ongoing medical management, education
Follow-up	1–2 times by surgeon	Ongoing for medical management, education
Role of specialist in Model of Care	Surgeon is the endpoint of the Model of Care	Rheumatologist is the beginning (gatekeeper) of the Model of Care
Types of teamwork	Sequential, multidisciplinary	Concurrent, interprofessional
Location of service delivery	Inpatient	Outpatient/Community

1. Purpose of Models of Care for Total Joint Replacement and Inflammatory Arthritis
  The overall purpose of the TJR models of care is to get appropriate surgical candidates to the surgeon for consult in a timely manner. Models of care for TJR are focused on an acute need with a single “fix” of surgical intervention. It is primarily a linear model of care with the surgeon and surgery the endpoint of care with health care professionals working sequentially with patients in a multidisciplinary approach to prepare patients for surgery. Specialist care is end-loaded and confined to a time-limited episode of care beginning with the surgical consult and ending with post-operative rehabilitation and follow-up.

*“…once they’ve had their surgery they are triaged according to their specific needs. So the majority of patients…go home without any further care… another group go home with home care; another group go home with outpatient physiotherapy; another group go to our fit program which is a short-term tune-up program for more complex patients. Then a select, smaller group would go to an inpatient rehab facility.”*

Unlike the TJR models of care, where the patient accesses the specialist (orthopedic surgeon) for a specific limited episode of care, in rheumatology, given the chronicity of Inflammatory Arthritis, patients need to continue to be seen and monitored by the rheumatologist across the trajectory of their condition. Inflammatory Arthritis is characterized by episodes of acute flares requiring timely medical assessment and intervention. So the practice rosters of rheumatologists can be filled with patients requiring follow-up and ongoing monitoring limiting the number of new patients that can be seen. Also, in contrast with TJR models of care where accessing the specialist can be seen as the end point, the rheumatologist is the beginning of the Inflammatory Arthritis model of care for diagnosis, initiation of medical management, and referral to the multidisciplinary team. Specialist care in Inflammatory Arthritis is front loaded and continues indefinitely either with the rheumatologist or in partnership with primary care physicians or Extended Role therapists. As a rheumatologist explains:

*“So if you have a severe rheumatic disease, you’re going to be on my books forever. I’m going to see you at least every 3 months ongoing. But if you have a limited issue, we’ll negotiate discharge back to primary care. And in most situations we’re in co-care with primary care.”*

Once seen by the rheumatologist, patients are typically referred for multidisciplinary assessment and education by team members such as nursing, physiotherapy and occupational therapy at minimum, and sometimes a dietician, pharmacist and/or social worker. All the Inflammatory Arthritis models of care have a multidisciplinary, outpatient program that provides assessment, education and self-management. These programs range from a 2 week, daily, intensive, personalized care program to occasional group education/clinic days. The goal of the majority of these programs is self-management skill development delivered either within the program or through community resources.

2. Follow-up
  Follow-up with TJR patients might be conducted by the orthopaedic surgeon, family physician, therapist from The Arthritis Society, or an Extended Role therapist. As one orthopaedic surgeon said,

*“We’re standardizing our follow-up protocol as well and we’re spreading out our follow-ups much more than we had in the past. So they’re seen two to three times within the first year and then at the 2 year mark, the 5 year mark, 10 year mark, and then, depending on how they’re doing beyond that, it’s a little bit more individualized.”*

In contrast, follow-up is a key component of the Inflammatory Arthritis model of care. It can be delivered by a variety of non-specialist health care professionals, in a variety of contexts such as, one on one, in person or in a group setting, or from a distance. Table 2 illustrates the various types of follow-up provided depending on issues such as group size and geographic constraints.

**Table 2:** Types of follow-up in inflammatory models of care

	One on one interaction	Group interaction
In person	Rheumatologist, PT, RN	Multi-disciplinary refresher days
Distance	Telemedicine, Telephone	Internet clinics, chat rooms

Inflammatory Arthritis models of care may include partnerships between rheumatologists and other practitioners such as Extended Role therapists or Registered Nurses.

*“Because once the diagnosis is made and a plan is put into place then there’s a fairly defined way of following and monitoring these patients so that you don’t have to have necessarily a physician involved at every stage.”*

In Alberta some rheumatologists have incorporated a Registered Nurse into the rheumatologist’s practice/drug monitoring program or clinic to follow-up patients who are on medications/biologics. For example, the pharmacovigilance program in Alberta for patients on biologic therapy records and monitors the long-term efficacy and adverse effects as well as measures the cost-effectiveness of biologics. The Registered Nurses are responsible for all the reassessments that are required to ensure renewal of the patients’ medications. In addition, they have the skills to perform intramuscular and intravenous injections and provide patients on such medications with instructions and teaching regarding these injections and/or arrangements to receive injections. Registered Nurses were described as being a valuable addition to the practice because they take a holistic approach to patient care addressing psychosocial issues and medication adherence problems that patients are experiencing. Moreover, the presence of a Registered Nurse can reduce the rheumatologist’s caseload e.g., by seeing stable patients at the 1-year mark instead of a visit with the rheumatologist.

*“Basically what ends up happening is that if a patient’s stable and they need to be seen only every 6 months, the rheumatologist will see them at 6 months and the pharmacovigilance nurse will see them at a year, so it really cuts our routine follow-ups in half.”*

In Ontario, some rheumatologists have partnered with Extended Role therapists. The Extended Role therapist may: 1) be given a subset of the rheumatologist’s caseload to follow, such as those who have established diagnoses, are stable, and on a chronic disease management pathway; 2) monitor activity or response to medications (e.g. effectiveness and side effects) to determine whether changes in treatment are required; and, 3) assess and recommend other resources that would help those individuals manage their arthritis. The Extended Role therapist may perform these follow-up assessments on days when the specialist is also available and/or in between the specialist’s visit in order to book those requiring a change in treatment regime in an upcoming clinic. Thus, the rheumatologist may not see all patients for a full review since the Extended Role therapist follows these stable patients independently but under supervision of the specialist. However, the rheumatologist is always involved with medication changes and other duties outside the Extended Role therapist’s scope of practice. At the time of data collection for this study, training of Extended Role therapists was only available in Ontario.

## Discussion

In this paper we set out to identify commonalities and differences in existing models of care for arthritis in three Canadian Provinces in order to identify contextual issues in the local implementation of these models. Canada provides a useful case given the combination of highly populated urban areas and underpopulated rural/remote areas that are difficult to access and require major travelling of both patients and health care professionals, all within publicly-funded settings.

Within the broad umbrella of models of care for arthritis we found two distinct models of care – one for TJR and one for Inflammatory Arthritis. Common issues in both models of care were lack of complete and appropriate referrals from primary care physicians and lack of health human resources to meet local demand, particularly in rural and remote areas. Local strategies to address these issues include standardized referrals and central/pooled intake, triage utilizing non-specialist arthritis health care professionals, and use of telemedicine and travelling clinics to bring specialists and patients together. Differences in the models of care for TJR and Inflammatory Arthritis included the rationale or reason for triage, the nature of the care or intervention provided (surgical vs medical), the nature and extent of follow-up, the role of the specialist, types of teamwork, and the location of service delivery (urban vs rural/remote).

Although the phrase “model of care for arthritis” is used in the literature, our findings indicate that there is not a single model of care for arthritis given the differences in disease trajectory and the availability of medical/surgical management for the various types of arthritis. Just the clinical needs of the different types of arthritis clearly lead to different models of care. Although the term “arthritis” is a common part of the English and medical vernacular, there is much less public awareness of the differing kinds of arthritis conditions (e.g. OA and Inflammatory Arthritis) and the differences in their management. The juxtaposition of these issues with demographic, geographic and health human resources issues, point to the need for health policy that is supportive of flexible, context-dependent, locally driven solutions.

Our findings illustrate how the focus of current models of care for arthritis is on the early medical management of Inflammatory Arthritis and TJR in the late stages of OA. Both represent areas in which there are currently accepted effective medical treatments (e.g. DMARDs and TJR) for the management of arthritis. Far less attention has been paid to the development of models of care for early to mid-stage OA, which accounts for the majority of cases of “arthritis”. This burden-service gap is not unique to Canada but is present in most developed nations [25]. The focus on acute Inflammatory Arthritis and late OA is probably due to the presence of medical technologies (drugs and surgery) for those two conditions resulting in clear, predictable pathways and targeted funding. In contrast, there is less effective medical management available to those with early to mid-stage OA. International models of care for OA typically use principles of chronic care management such as care coordination, multidisciplinary team interventions and collaborative care planning with persons with OA [25]. Even though TJR is only part of the overall management of OA, the models of care in this study are focused on the terminal stages of the condition when surgery is indicated. We found few (if any) organized models of care for people with OA who are not considered appropriate for surgery. Rather, these patients are dependent on haphazard availability of community-based services with unclear funding models and challenges in coordination and continuity between and amongst service providers. These findings highlight the care gap for those with non-inflammatory, non-surgical arthritis, who represent the highest numbers of people with arthritis. These findings will be presented further in a later publication.

Our findings indicate that reliance on primary care providers as gatekeepers to specialist care can be problematic given the growing and changing complexity of healthcare demand. Considerable time and resources have been expended in the existing models of care for arthritis we identified on improving the information received from primary care physicians in order to ensure timely and appropriate access to specialist services. Research has shown that primary care physicians by and large are ill-equipped to diagnose and manage musculoskeletal conditions such as early to mid-stage OA [24, 26]. Yet there are other health care professionals with considerable expertise in the assessment and management of chronic musculoskeletal conditions.

Our findings show how local need for health human resources has led to innovative models of care for arthritis that incorporate the skills of other non-specialist health care professionals such as Registered Nurses and Extended Role therapists to help stream and triage the referral process. However, only in Ontario are non-specialist health care professionals working in Extended Role roles in rheumatology and TJR settings. Research has shown models using extended role practitioners are acceptable to patients [27] and that a higher proportion of patients receive TJR when triaged to see the orthopaedic surgeon [28–30]. Provincial legislation governs the scope of practice of health care professionals in each province and while Ontario has committed to ensuring that health care professionals are working to their maximum scope of practice and is working with regulatory bodies to amend legislation [19], British Columbia and Alberta have less such activity. Ongoing and projected workforce shortages [22, 31–34] will require that health care professionals work together in new ways to provide care to a growing number of people with arthritis.

The differences in the utilization of non-specialist arthritis health care professionals in the TJR models of care as opposed to the Inflammatory Arthritis models of care points to how scope of practice and funding can drive the models. In Canada, universal health coverage under Medicare only includes hospital-based services and physician services. Because TJR occurs in hospital settings, funding is available for non-specialist health care professionals in extended roles. In contrast, management of Inflammatory Arthritis is mainly community-based and therefore funding for non-specialist health care professionals is tied to the rheumatologists’ billing, driving up costs and decreasing the availability of scarce rheumatology resources by ensuring that all patients must be seen by a specialist.

The availability of health human resources with respect to specialist care for arthritis is an international issue. In a recent review, Badley and Davis [23] identified the following issues as key to the ability to provide necessary specialist care for arthritis both now and in the future: 1) inadequate availability of rheumatologists and orthopaedic surgeons to meet needs for care of projected numbers of people with arthritis; and 2) the focus of current specialist care on Inflammatory Arthritis and TJR ignores the majority of the population with arthritis creating a major gap in care delivery. Models of care for arthritis are required that maximize the appropriate use of specialists and other health care professionals with extended skills and training.

There are limitations to this paper that need to be acknowledged. It should be noted that this research was intended to identify existing models of care for arthritis in three provinces, not every individual model of care for arthritis in each province. As such, there may be existing models of care for arthritis that we have not identified that may address some of the issues identified above. Some might argue that we did not find different models of care for arthritis; rather, we found different service delivery locations that have modified the traditional primary care physician-specialist model of care. As such, our paper describes modifications to the existing, traditional models of care, rather than unique models of care for arthritis. This argument highlights the challenges with the term “models of care” that have been identified elsewhere [12]. Issues of terminology notwithstanding, our findings demonstrate commonalities and differences in the ways that different locations of service delivery have modified traditional models of care for persons with arthritis that have implications for health policy and service delivery.

## Conclusions

We have identified numerous existing models of care in Canada for people with arthritis, the majority of which are focused on TJR and Inflammatory Arthritis, with few, if any, structured programs of care for persons with non-inflammatory, non-surgical arthritis. There continues to be barriers to receiving timely care and care gaps for some patient groups, particularly those for which there are not well-developed medical interventions with clear funding paths. Future work in developing and implementing models of care for arthritis needs to consider the overall population with arthritis, the continuum of care, and utilize the best components of these often locally-developed models to provide coordinated, equitable and effective care by the available professional and community resources.

## Additional files

Additional file 1: Semi-structured intial Interview Guides. (DOC 44 kb) Additional file 2: Semi-structured follow-up Interview Guides. (DOC 48 kb) Additional file 3: Consent Information Letters. (DOC 326 kb) Additional file 4: Scripts for obtaining consent. (DOC 45 kb)

## Abbreviations

DMARDs: Disease Modifying AntiRheumatic Drugs
OA: Osteoarthritis
TJR: Total Joint Replacement
