# Supplementary material for: Commonalities and differences in the implementation of models of care for arthritis: key informant interviews from Canada
Source: BMC Health Serv Res. 2016 Aug 19;16:415. doi: 10.1186/s12913-016-1634-9 (PMC4992288; doi:10.1186/s12913-016-1634-9)
Supplement: Additional file 1: — Semi-structured intial Interview Guides. (DOC 44 kb) [file 12913_2016_1634_MOESM1_ESM.doc]

# Key Informant Interview Guide – Initial Interview

| **Date: ____________________** | **Interviewer: ________________________________** |
| --- | --- |
| **Title/Position: _____________________________________________________________** | |
| **Model Type: ______________________________________________________________** | |

**Background**

We are interested in understanding how care is organized for people with arthritis. Some people refer to this as a model of care. Your organization has been identified as one that has specifically addressed care for people with arthritis. We would like to ask you some questions about how the care is organized so that we can understand your model of care.

# It would be helpful to start by understanding a bit about your role and the setting. Then, I’ll ask you some questions about how care is organized for people with arthritis.

**Interview Questions**

1. **Briefly describe your position and experience related to arthritis care.**

Role

Setting

Years Experience

Education

1. **Can you tell me about how care is organized for people with arthritis?**
   - should describe the model

Who is the target population? *(probes: type of arthritis, stage of disease or focus of intervention)*

What services/care are provided/available? *(probes: type, where delivered)*

Who delivers interventions? *(probes: professions)*

How do people access the model of care? *(probes: referral process)*

- How long does it take for a patient to be seen from the time they are referred by a doctor?

How does the process work? *(probes: Could you walk me through care as if I were a patient?)*

- How would I re-access care if I needed to come back?

What are the communication processes? *(probes: among the team members; to referring physician; for onward care such as to community services, etc.)*

Are quality measures used? For example, what process measures are used? What outcome measures? (Outcomes might be for the program e.g., staff satisfaction or they might be patient outcomes)

- If you have quality measures are you asking patients to evaluate the services?

Yes / No

1. **When was the model established?**

Thank you for answering my questions.

1. **Is there anyone else you think I should talk to in your organization related to this topic?**

This is part one of our project. The second part will involve a more detailed interview related to different aspects of how arthritis care is managed. We will only be re-contacting some of the people we have talked to as we will focus on specific models of care. We will not know which models will be explored in more detail until we have completed these first interviews.

**5) Would you be willing to talk to us again?**

**Yes / No**

If not, is there another person in your organization who we might contact?

**6) Are you aware of other organizations where there are organized models of care related to arthritis?**

If, yes, who would be the best contact person?
