# Supplementary material for: Commonalities and differences in the implementation of models of care for arthritis: key informant interviews from Canada
Source: BMC Health Serv Res. 2016 Aug 19;16:415. doi: 10.1186/s12913-016-1634-9 (PMC4992288; doi:10.1186/s12913-016-1634-9)
Supplement: Additional file 2: — Semi-structured follow-up Interview Guides. (DOC 48 kb) [file 12913_2016_1634_MOESM2_ESM.doc]

# Key Informant Interview Guide – Follow-Up Interview

| **Date: ____________________** | **Interviewer: ________________________________** |
| --- | --- |
| **Title/Position: _____________________________________________________________** | |
| **Model Type: ______________________________________________________________** | |

**Background**

We are interested in understanding how care is organized for people with arthritis. Some people refer to this as a model of care. Your organization has been identified as one that has specifically addressed care for people with arthritis. We previously asked you about how care was organized for people with arthritis. We’d quickly like to recap to make sure we have understood that information correctly. Then we would like to ask you some further questions about the drivers of developing the model of care in this way and what you feel are the challenges related to care delivery for people with arthritis.

**Recap Questions**

# I’ll start by confirming nothing has changed with the information I have.

# 1) Confirm position, role, background, etc.

# 2) When we talked previously, you described care for people with arthritis in your setting in the following way. Can you confirm that I have this correct?

**Interview Questions**

**3) What was/were the driving factor(s) behind the development and implementation of the model (e.g., governmental, local institution, etc.)?**

**4) How was the type of model or its characteristics determined?**

What data were used to support the type of model?

What aspects of the local situation drove the model (e.g., need, resources, etc.)?

**5) What is absolutely necessary for the model to work, both tangibles and intangibles (e.g., health professionals, institutional support, etc.)?**  *(probes: at a macro, messo or micro level)*

**6) What do you see as constraints or difficulties related to the model?** *(probes: personnel, funding, inter-personal relationships, allowed scope of practice, patient volumes, access issues such as geographical, health care provider shortages, direct access)*

How can these challenges be overcome?

**7) Do you perceive that the model has sufficient flexibility to change as needed in the future?**

1. **Has the model been evaluated?** Yes / No

If so, what aspects and what were the findings? *(probes: system impact, process indicators, patient outcomes, etc.)*

**9) If you were to do this again, what would you do differently?** *(probes: re to address gaps in current model, what would you change/enhance/diminish; perception of need to broaden continuum of care addressed, etc.)*

**10) If you were to give advice to someone about setting up a model of care, what would that be?** *(probes: key elements, etc.)*

***Other questions to ask if occasion arises:***

11) How have other stakeholders interpreted your development of the model?

12) What is your opinion on the concept that by developing a specific model, it ‘cannibalizes’ resources away from other services?

13) Were there trade-offs in developing this model? In other words, by implementing this model have you had to reduce services elsewhere?

14) To what extent has the current funding model (either for hospitals, physicians, and others) held or hindered your model?

15) Do you think that increasing the scope of practice for non-physician providers would facilitate care provided in the model? If yes, then how?

**Thank you for taking the time to speak with us today.**

**16) Would you be willing to be contacted if we require any clarifications once we analyze our data?**

Yes / No
