# Supplementary material for: Commonalities and differences in the implementation of models of care for arthritis: key informant interviews from Canada
Source: BMC Health Serv Res. 2016 Aug 19;16:415. doi: 10.1186/s12913-016-1634-9 (PMC4992288; doi:10.1186/s12913-016-1634-9)
Supplement: Additional file 3: — Consent Information Letters. (DOC 326 kb) [file 12913_2016_1634_MOESM3_ESM.doc]

###
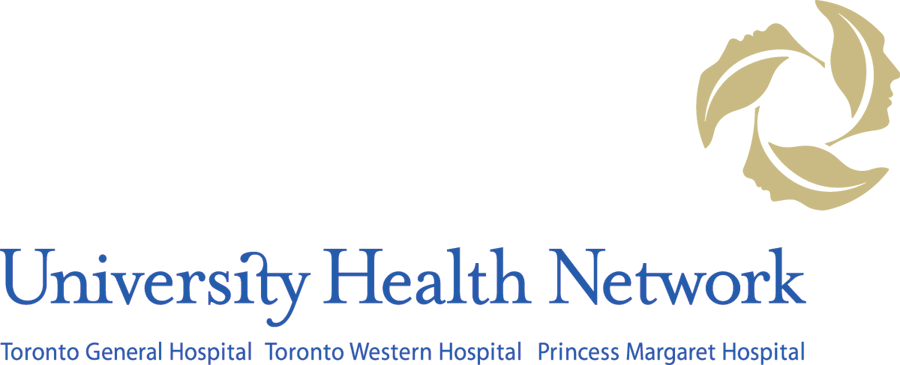


### CONSENT INFORMATION LETTER

# Models of Care in Arthritis, Bone & Joint Disease (MOCA)

| Principal Investigator: | Dr. Aileen Davis, Ph.D. Division of Health Care and Outcomes Research  and Arthritis Community Research and Evaluation Unit Toronto Western Research Institute  MP 11-322, 399 Bathurst Street  Toronto, ON M5T 2S8  Phone: (416) 603-5543 |
| --- | --- |
| Research Team: | Elizabeth Badley (Co-Investigator), Kathleen Bednis (Research Associate), Louise Bergeron (Co-Investigator), Cheryl Cott (Co-Investigator), Cy Frank (Co-Investigator), Gillian Hawker (Co-Investigator), Allyson Jones (Co-Investigator), Mike Landry (Co-Investigator), Linda Li (Co-Investigator), Crystal MacKay (Trainee), Rose Wong (Study Coordinator) |

# Sponsor: Canadian Institutes of Health Research

# You are being asked to take part in a research study. Please read this explanation about the study and its risks and benefits before you decide if you would like to take part. You should take as much time as you need to make your decision. You should ask the study investigator or study staff to explain anything that you do not understand and make sure that all of your questions have been answered. Participation in this study is voluntary.

# Background and Purpose

# Using existing research and knowledge of experts in the field, we hope to generate an evidence-based framework that can be used by health care professionals and policy- and decision-makers within the health care system to develop a comprehensive model of arthritis care that includes a toolkit for adopting/implementing a model that fits within their context. It will enable people with arthritis to be active partners in accessing appropriate care at the right time, by the right provider, over the course of their disease. Our research will consider factors potentially influencing the type of models of care that jurisdictions choose to implement including population needs, health system factors, geographical issues and characteristics of current models of care. Participants will be selected who represent/can provide insight on current models of care for arthritis/musculoskeletal conditions, as well as including those within the context of chronic disease management more generally.

# Study Procedures

Participation in this study will involve being interviewed and audio-taped. The interviewer will be asking questions about your role and experience related to arthritis care and how care is organized for people with arthritis. Interviews will take approximately 45 minutes to one hour and will be conducted by telephone at a time that is convenient for you.

This is part one of our project. The second part will involve a more in-depth, follow-up interview related to the ‘how’ and ‘why’ models were developed. These interviews will also take approximately 45 minutes to one hour. We will only be re-contacting some of the people we have talked to as we will focus on specific models of care. You will be asked during this first interview whether you would be willing to talk to us again. However, we will not know which models will be explored in more detail until we have completed these initial interviews.

Once again, these interviews will be audio-taped to allow us to review the details of the interview and to analyze the information you provide. The audiotapes will be sent to an external agency and will be transcribed verbatim.

# Risks Related to Being in the Study

There are no anticipated risks from being in this study.

# Benefits to Being in the Study

# You will not receive any direct benefit from being in this study. However, information learned from this study will contribute to understanding models and delivery of arthritis care across the continuum, leading to an innovative decision-making framework and toolkit for enhancement of care.

Voluntary Participation

Your participation in this study is voluntary. You may decide not to be in this study, or to be in the study now and then change your mind later. You may leave the study at any time. You may refuse to answer any question you do not want to answer, or not answer an interview question by saying ‘pass’.

Confidentiality

All information obtained during the study, including your personal health information, will be kept confidential and will not be shared with anyone outside the study unless required by law. Names will never be used in the findings reported from this study. No one except research staff will have access to details of the information collected.

Only the audiotapes will be sent to an external agency for transcription. No other information that directly identifies you will be transferred outside the investigators in this study or this hospital. The tapes and transcripts will be stored and secured in a locked cabinet at the research office to which only the researchers have direct access. Tapes will be destroyed once a transcript becomes available. Transcripts will have a code and will not show your name or address.

If you decide to leave the study, your information that was collected before you left the study will still be used. No new information will be collected without your permission.

**Questions About the Study**

If you have any questions, concerns or would like to speak to the study team for any reason, please contact the person in charge of this study, Dr. Aileen Davis at number 416- 603-5543 or email [adavis@uhnresearch.ca](mailto:adavis@uhnresearch.ca). You may also contact the Study Coordinator, Rose Wong at number 416-603-5665 or toll free at 1-877-818-7340 or email [rwong@uhnres.utoronto.ca](mailto:rwong@uhnres.utoronto.ca).

If you have any questions about your rights as a research participant or have concerns about this study, call the Chair of the University Health Network Research Ethics Board (REB) or the Research Ethics office number at 416-946-4438. The REB is a group of people who oversee the ethical conduct of research studies. These people are not part of the study team. Everything you discuss will be kept confidential.

## **Consent**

## By participating in the interview, you are giving voluntary consent to take part in this study.
