# Supplementary material for: Commonalities and differences in the implementation of models of care for arthritis: key informant interviews from Canada
Source: BMC Health Serv Res. 2016 Aug 19;16:415. doi: 10.1186/s12913-016-1634-9 (PMC4992288; doi:10.1186/s12913-016-1634-9)
Supplement: Additional file 4: — Scripts for obtaining consent. (DOC 45 kb) [file 12913_2016_1634_MOESM4_ESM.doc]

# Models of Care in Arthritis, Bone & Joint Disease (MOCA):

# Telephone Script & Verbal Consent

# *[Use if voice mail]*

Hello, my name is {insert name}. I’m the {insert position} on the project, Models of Care in Arthritis, Bone & Joint Disease, otherwise known as MOCA. {I’m calling from Toronto, Ontario}. You have been identified as someone whose perspective could greatly inform this study regarding models of service delivery for people with arthritis/MSK conditions. You would have received an information letter via email a couple of days ago regarding this study. Please give me a call at 416-603-5665 (or toll free 1-877-818-7340). Our office hours are {insert time}. Thank you.

# *[If two attempts by phone and messages left both times with no response from potential participant, then use email]*

**Subject:** Aileen Davis/{Insert Co-I} – Potential Key Informants for New Study, Models of Care in Arthritis, Bone & Joint Disease (MOCA)

Dear {insert potential participant’s name}

You have been identified as someone whose perspective could greatly inform this study regarding models of service delivery for people with arthritis/MSK conditions. I’m the {insert position} on the project, Models of Care in Arthritis, Bone & Joint Disease, otherwise known as MOCA. I’m following up on the information letter which you would have received via email a couple of days ago regarding this study. I would like to talk to you about this research, would it be possible to set up a time when we can speak over the phone.

Thank you!

# *[If no response by email assume individual is not interested in participating]*

# *[Use if speaking to a respondent]*

Good morning/afternoon. You have been identified as someone whose perspective could greatly inform this study regarding models of service delivery for people with arthritis/MSK conditions. I’m the {insert position} for the study. Do you have some time for me to tell you about the research?

If ‘no’, what would be a convenient time to call you back?

| _________________________ | _________________________ |
| --- | --- |
| **Date** | **Time** |

If ‘yes’, then proceed to discuss the study.

Before I begin, I was wondering whether you have had a chance to review the information letter that was sent to you about a week ago?

The study brings together investigators from ON, BC and AB *{list province in order so that first province is the one being called}*. This is a 5-year emerging team grant funded by CIHR. The research office is located at the TWH in ON.

The data we are gathering from all sources will be used in developing an evidence-based framework and toolkit that will guide health care practitioners and policy/decision-makers within the health care system through key considerations in creating and implementing a model of care. It will assist them in making an informed decision regarding the type and resourcing required for a viable model within their specific context.

This study involves a telephone interview that takes approximately 45 minutes to one hour to complete. The interviewer will be asking questions about your role and experience related to arthritis care and how care is organized for people with arthritis. We want to know about the type and characteristics of the model of care that is taking place at *{insert place/organization}*. A date and time for the interview can be arranged that is most convenient for you.

This is part one of our project. The second part will involve a more in-depth, follow-up interview related to the ‘how’ and ‘why’ models were developed. We will not know which models will be explored in more detail and who we will need to re-contact until we have completed these initial interviews.

Interviews will be audio-taped and transcribed verbatim.

Your participation in this study is voluntary. You may decide not to be in this study, or to be in the study now and then change your mind later. You may refuse to answer any question you do not want to answer.

All information collected during this study will be kept confidential and will not be shared with anyone outside the study. Only the audiotapes will be sent to an external agency for transcribing. We will never use any names when reporting the findings of this study. The tapes and transcripts will be secured in a locked cabinet at the research office to which only the research team has access. The tapes will be destroyed once a transcript becomes available.

Our research team is lead by Dr. Aileen Davis and my name is {insert name}. If you have any questions, concerns, or would like to speak to the study team for any reason, I will give you our contact numbers in a moment.

This research is overseen by an ethics board at the University Health Network. If you have any questions about the ethical conduct of research at the hospital including your rights as a research participant, you may contact the Chair of the Research Ethics Board. I will also give you the contact number in a moment.

Do you have any questions about the study?

**Do you agree to take part in this study?**

**Participant’s Verbal Consent to Interview: Yes / No**

**If ‘no’.** Thank you for considering our request. Thank you for your time. *[End call]*

**If ‘yes’.** Great! *[Proceed to complete verbal consent process and obtain interview date]*

| _________________________ | _________________________ | __________________ |
| --- | --- | --- |
| **Print Name of Person Obtaining Consent** | **Signature** | **Date** |

**Do you consent to being audio-taped?**

**Participant’s Verbal Consent to Audiotape: Yes / No**

**If ‘yes’**

| _________________________ | _________________________ | __________________ |
| --- | --- | --- |
| **Print Name of Person Obtaining Consent** | **Signature** | **Date** |

The phone numbers that I’d mention above are available on the information letter that you have previously received by email. Would you still like to write down these contact numbers?

**If ‘yes’**. You can reach Dr. Aileen Davis, myself, or other members of the study team at 416- 603-5665 or 1-877-818-7340. If you have concerns about your rights as a participant in this study, feel free to call the REB office at 416-946-4438.

By participating in the interview, you are giving consent to take part in this study. If convenient for you, we can go ahead and arrange a date and time for the interview. Once again, it will take approximately 45 minutes to one hour.

**When would you like to schedule the phone interview?**

| _________________________ | _________________________ |
| --- | --- |
| **Interview Date** | **Interview Time** |

If you need to reschedule please don’t hesitate to contact me. I will send an email a few days prior to our scheduled time to confirm our interview. Thank you very much for your time!

**ID#: _________________**
